# Clinical inertia in cardiovascular risk management: prevalence, associated factors, and impact on outcomes of the OPM study

**DOI:** 10.7189/jogh.16.04256

**Published:** 2026-09-11

**Authors:** José Abellán Alemán, Pablo Sánchez-Rubio Lezcano, Daniel Escribano Pardo, Luis Castilla Guerra, Rafael Crespo Sabaris, Guillermo Jiménez Portillo, Begoña Rincón Ruiz, Gloria Antón Pérez, Jesús Iturralde Iriso, Pilar Segura, Pedro J Tárraga López, Javier Nieto Iglesias, José Francisco López-Gil, Researchers of the OPM Study, José Abellán Alemán, Javier Nieto Iglesias, Pedro J Tárraga López, Francisco José Fuentes Jiménez, Fernando García Romanos, Jesús Palomo del Arco, Alcibíades Segundo Díaz Vera, María Dolores Martínez Esteban, Alfonso Pobes Martínez de Salinas, Pablo González Bustos, Luis Castilla Guerra, Guillermo Jiménez Portillo, Emilio Márquez Contreras, Pilar Segura Torres, Ana Palomo Ruiz, Nicolás José Quero Fernández, María Cristina Uclés Peña, Alicia Tébar Vizcaíno, Sergio García González, Irene Fernández Carreño, Marina Cejudo Casas, María Esther Salguero Cámara, Sara Márquez Rivero, María Victoria Quesada Lara, Julio López Fernández, Cinthya Carolina López, Olga Vargas Gómiz, Dulce Martínez Cámara, Estefanía Ríder Reyes, Pablo Sánchez-Rubio, Cristina Lueza Lampurlanes, María Lalueza Cosculluela, Laura Hernández Camacho, Ana Belén Solano Checa, Ana Isabel Grau Barrull, Maria Victoria Coiduras Sanagustin, Daniel Escribano Pardo, Ignacio Durán Sánchez, Lucia Cásedas Aguarón, Eva María Samatán Ruiz, Ana Cristina Martínez Sancho, Rafael Crespo Sabaris, Belén Hernández Ledesma, Laura Moreno Fernández, Esther Nieva Porres, Isabel Sancasimiro Canzano, Lourdes Canta Castro, Beatriz López Santa María, Ángeles Velasco Soria, Esther Uceda Gómez, Ángeles Robles Reyes, José Basilio Gómez Castaño, Francisco Lafuente Salanova, Juan Francisco Martínez García, María José Martí Montoya, Ramón López Guillén, Florentina Rosique, Daniel Sáenz Martínez, Jesús Iturralde Iriso, Olga López López, Amaya Arrieta Aguado, Beatriz Barrios Núñez, Aránzazu Villa Sanabria, Alba Gamboa Serrano, Esperanza Moral Berrio, Susana Pedrajas Molina, Patricia Sánchez Escudero, Francisco Javier Alonso Moreno, María Dolores Martínez Malabia, Begoña Rincón Ruiz, José Antonio Navarro Fernández, Andrea Torres Calatayud, Belén Almudena Tárraga Marcos, Francisco Javier Lucas Pérez-Romero, Deyse Helena dos Santos, Francisca Molina Escribano, Carlos Jesús Cabezas Reina, Laura Cueto Bravo, Borja Alonso Calle, María Ibáñez Cerezo, María Antonia García Rubiales, Marta Romero Molina, Francisco Javier Ahijado Hormigos, Isabel Hervella Durantez, Esperanza González Álvaro, Greta Asunción Spairani Martínez, Juan Pablo Sánchez Núñez, Fernando Martínez García, Pablo Rodríguez Doyagüez, Arianne Aiffil Meneses, Jorge Valdés Sotomayor, Laura Vanessa Blanco Andrews, Emilsi Trinidad Pérez, Freddy Guzmán Ames, Diego Fabián Sidel, José Valderrama Marín, Mª Victoria Guijarro Abad, Enrique Alberto Ruíz Donoso, José Antonio Gómez Puerta, Aurea Mauren Guevara Bustamante, Adriana Lizeth Galindo Guarnizo, Gloria Antón Pérez

**Affiliations:** 1 Cátedra de Riesgo Cardiovascular, Universidad Católica de Murcia, Murcia, Spain; 2 Servicio de Medicina Interna, Hospital San Jorge, Huesca, Spain; 3 Centro de Salud Oliver, Zaragoza, Spain; 4 Servicio de Medicina Interna, Hospital Clínico Universitario Virgen Macarena, Sevilla, Spain; 5 Centro de Salud de Entrena, La Rioja, Spain; 6 Servicio de Nefrología, Hospital Universitario Poniente, Almería, Spain; 7 Servicio de Nefrología, Hospital de Cuenca, Cuenca, Spain; 8 Centros de Diálisis, Avericum, Spain; 9 Centro de Salud Aranbizkarra, Vitoria, Spain; 10 Servicio de Nefrología, Hospital de Jaén, Jaén, Spain; 11 Faculty of Medicine, University of Castilla-La Mancha, Albacete, Spain; 12 Gerencia de Atención Integrada de Albacete, Servicio de Salud de Castilla-La Mancha, Albacete, Spain; 13 Servicio de Nefrología, Hospital General Universitario, Ciudad Real, Spain; 14 School of Medicine, Universidad Espíritu Santo, Samborondón, Ecuador; 15 Faculty of Health Sciences, Universidad Autónoma de Chile, Temuco, Chile

**Keywords:** primary care, therapeutic intensification, guideline adherence, cardiovascular prevention, treatment optimisation, quality of care

## Abstract

**Background:**

Clinical inertia, defined as the failure to initiate or intensify therapy when clinically indicated, represents a major and under-addressed barrier to achieving cardiovascular risk (CVR) factor control in primary care. Despite the availability of evidence-based guidelines and effective pharmacological treatments, many patients with uncontrolled hypertension, diabetes, or dyslipidaemia do not receive appropriate treatment intensification. We aimed to evaluate the prevalence of clinical inertia, identify associated patient-level factors, and assess its impact on CVR factor outcomes in high-risk primary care patients.

**Methods:**

This was a pre-specified secondary analysis of the *Objetivo Punto de Mira* (OPM) study, a multicentre, prospective, quasi-experimental interventional study conducted in primary care settings across nine Spanish regions (2024–2025). We included 711 participants with at least one uncontrolled CVR factor at baseline (hypertension, diabetes, or dyslipidaemia). Clinical inertia was operationalised, among therapeutically adherent patients, as the absence of documented pharmacological treatment initiation, dose escalation, or appropriate combination therapy when a risk factor remained uncontrolled. We estimated prevalence with 95% confidence intervals (CIs). Multivariable logistic regression identified factors associated with clinical inertia. Linear mixed-effects models assessed changes in CVR factors from baseline to 90-day follow-up, comparing patients with and without clinical inertia. Risk analysis quantified the association between clinical inertia and persistent uncontrolled status.

**Results:**

Among patients with uncontrolled conditions at baseline, clinical inertia was observed in 49.3% (n/N = 208/422) for hypertension, 31.1% (n/N = 73/235) for diabetes, and 38.2% (n/N = 217/568) for dyslipidaemia. Overall, 54.3% of participants (n/N = 386/711) exhibited clinical inertia in at least one condition. We found no statistically significant associations between clinical inertia and age, sex, CVR category, or excess weight. Clinical inertia significantly increased the risk of remaining uncontrolled across any condition (relative risk = 1.13; 95% CI = 1.03, 1.24; absolute risk difference = 9.3%; 95% CI = 2.4, 16.2; number needed to harm ≈ 11, *P* = 0.007), though not for individual conditions separately. At 90-day follow-up, patients with clinical inertia showed significantly smaller reductions in total cholesterol (between-group difference in change = 19.3 mg/dL; 95% CI = 12.4, 26.3, *P* < 0.001) and low-density lipoprotein cholesterol (15.5 mg/dL; 95% CI = 8.9, 22.0, *P* < 0.001) compared with patients who received treatment intensification. We also observed a small but statistically significant difference in body mass index reduction (0.25 kg/m^2^; 95% CI = 0.02, 0.49, *P* = 0.036). Both groups showed significant within-group reductions in systolic and diastolic blood pressure (*P* < 0.001); however, the magnitude of change did not differ significantly between patients with and without clinical inertia (between-group interaction *P* > 0.05 for systolic blood pressure and diastolic blood pressure). We found no significant between-group differences for glycaemic parameters, high-density lipoprotein cholesterol, or triglycerides.

**Conclusions:**

Clinical inertia affects more than half of high-risk primary care patients with uncontrolled CVR factors. Patient demographic and clinical characteristics examined (age, sex, CVR category, excess weight) did not predict clinical inertia in this sample, consistent with, but not proof of, provider- and system-level influences described in prior literature.

Cardiovascular diseases (CVDs) remain the leading cause of disability-adjusted life years (DALYs) and premature mortality worldwide. CVDs accounted for approximately 437 million DALYs globally in 2023, representing a 1.4-fold increase compared with 1990 [1]. Despite evidence-based guidelines and effective pharmacological interventions, a substantial proportion of patients with established cardiovascular risk (CVR) factors fail to achieve recommended therapeutic targets [2,3]. This persistent gap between guideline recommendations and clinical reality constitutes one of the foremost challenges in contemporary cardiovascular prevention. Failure to reach recommended therapeutic targets reflects distinct mechanisms, including patient non-adherence, treatment-resistant disease, suboptimal regimens, and clinical inertia. In our analysis, we focused on the latter, operationalised through documented prescribing behaviour in therapeutically adherent patients.

While medication non-adherence has received considerable attention, clinical inertia has emerged as an equally consequential, yet comparatively neglected obstacle to optimal CVR management [4,5]. The concept of clinical inertia was formally introduced by Phillips *et al.* in 2001, who defined it as the failure of healthcare providers to initiate or intensify therapy when indicated [6]. It has since been extensively documented across multiple chronic conditions, including hypertension, diabetes, and dyslipidaemia [7–10].

In hypertension management, clinical inertia has been reported in 30–50% of patients with uncontrolled blood pressure [11,12]. Comparable rates have been observed in diabetes care, where therapeutic inertia affects 20–40% of patients with suboptimal glycaemic control [13,14], and in dyslipidaemia management, with inertia rates ranging from 25–45% [15,16]. The consequences extend beyond poor risk factor control, with clinical inertia being associated with higher rates of cardiovascular events, hospitalisations, and mortality, as well as increased healthcare costs [17–20].

Despite this evidence, several important knowledge gaps remain. First, most existing studies have focused on individual conditions in isolation, with limited data on the simultaneous occurrence of inertia across multiple CVR factors [21]. Second, the patient- and system-level determinants of clinical inertia are incompletely characterised, with inconsistent findings across studies [22,23]. Third, real-world primary care evidence on the intermediate-term consequences of inertia, where most CVR management takes place, remains scarce [24].

The *Objetivo Punto de Mira* (OPM) study provided an opportunity to address these gaps. The primary objectives of our analysis were to estimate the prevalence of clinical inertia for each CVR factor separately, identify patient-level factors associated with inertia, and evaluate the impact of clinical inertia on CVR factor outcomes at 90-day follow-up.

## METHODS

### Study design and setting

We conducted a pre-specified secondary analysis of the OPM study, a multicentre, prospective, quasi-experimental interventional study under routine clinical practice conditions in primary care settings across nine Spanish regions between 2024 and 2025. We reported this secondary analysis in accordance with the STROBE guidelines (Table S1 in the **Online Supplementary Document**).

### OPM study intervention and implications for secondary analysis

The OPM study was a prospective interventional study conducted in ambulatory care across Spanish National Health System primary care centres and hospital outpatient clinics (2024–2025), approved by regional research ethics committees (Table S2 in the **Online Supplementary Document**). As stated in the approved patient information sheet, the study aimed to: quantify the proportion of high- and very-high-risk patients outside guideline targets for blood pressure, lipids, and glycaemia; distinguish therapeutic non-adherence from inadequate or insufficient treatment; and correct therapeutic inertia by reinforcing adherence in non-compliant patients and, among adherent but uncontrolled patients, intensifying treatment to the maximum tolerated drug doses and/or appropriate pharmacological combinations to achieve medium-term control.

In the OPM study, adherence at baseline was assessed using a structured questionnaire (Haynes–Sackett). Among ambulatory participants, 140 (17.8%) were non-adherent at baseline; 114 became adherent after standardised reinforcement, and 26 (18.5% of initially non-adherent patients) withdrew from the study for persistent non-adherence. A separate cohort of 501 dialysis patients was studied independently and was excluded from our analysis because their care organisation and treatment pathways differ materially from those of the study population.

The structured OPM protocol and monthly follow-up may have increased treatment vigilance (Hawthorne effect), potentially underestimating the prevalence of clinical inertia compared with unstructured practice. We did not design our analysis to test a dedicated anti-inertia intervention (*e.g.* audit-and-feedback targeting therapeutic passivity).

### Patient recruitment and eligibility assessment

In the OPM study, participating physicians were instructed to consecutively screen and recruit eligible patients presenting for routine outpatient consultations at their primary care practices. Eligibility was assessed prospectively by the treating physician at baseline. Eligibility criteria were verified against the patient’s electronic medical record (EMR), supplemented by direct clinical assessment where necessary.

Patients were eligible for inclusion if they were adults (≥18 years) classified as having high or very high CVR according to the 2021 European Society of Cardiology (ESC) guidelines on cardiovascular disease prevention [25]. High or very high CVR was defined by the presence of at least one of the following criteria: documented established CVD; target organ damage, defined as type 2 diabetes mellitus with duration >10 years (ascertained from the date of first diagnosis recorded in the EMR) or chronic kidney disease (estimated glomerular filtration rate (eGFR) <60 mL/min/1.73 m^2^); severe risk factor elevation, specifically low-density lipoprotein cholesterol (LDL-c) >190 mg/dL or grade 3 hypertension; or a calculated 10-year fatal CVR of ≥5% (high risk) or ≥10% (very high risk) based on the Systematic COronary Risk Evaluation 2/SCORE-Older Persons algorithm.

Exclusion criteria, including active collagenopathies, current immunosuppressive therapy, severe hepatic insufficiency (transaminases >2 × the upper limit of normal), pregnancy or lactation, life expectancy <1 year, and inability to provide informed consent, were verified through the EMR, specifically *via* medication records, active problem lists, and recent laboratory results. Self-reported data were cross-referenced with EMR entries where feasible.

In our analysis, we included all OPM participants who completed the baseline assessment and had at least one uncontrolled CVR factor at baseline.

### Study assessments and data collection

The OPM study included two scheduled assessments: baseline and ~90 days follow-up. The attending physician collected all study data using a standardised electronic case report form (eCRF). Sociodemographic data (age, sex) and self-reported information (treatment history, lifestyle habits) were obtained directly from the patient during the clinical encounter. Clinical measurements (blood pressure, anthropometry) were performed at the practice by trained healthcare personnel following standardised protocols. Laboratory biochemical parameters were retrieved from the patient’s EMR.

### Clinical measurements

#### Blood pressure

Office blood pressure was measured at each assessment time point in accordance with the 2018 ESC/European Society of Hypertension (ESH) guidelines for the management of arterial hypertension [26]. Measurements were performed using validated semi-automatic oscillometric devices, with patients seated and at rest for at least five minutes beforehand. Three consecutive measurements were taken at one-minute intervals; the mean of the last two readings was recorded as the assessment blood pressure value. This approach, which discards the first reading to account for the alerting response and averages the subsequent two to minimise random measurement error, is consistent with the recommendations of the 2018 ESC/ESH guidelines and is widely used in clinical research [26].

#### Anthropometry

Body weight and height were measured using calibrated standardised equipment, with participants wearing light clothing and no shoes. Body mass index (BMI) was calculated as weight (kg) divided by height squared (m^2^). Excess weight was defined as BMI ≥ 25 kg/m2.

#### Laboratory parameters

Biochemical data, including lipid profile (total cholesterol (TC), LDL-c, high-density lipoprotein cholesterol (HDL-c), and triglycerides (TG)), fasting plasma glucose (FPG), and glycated haemoglobin (HbA1c), were retrieved from the patient’s EMR. For baseline assessments, laboratory values obtained within three months prior to baseline were accepted. When multiple results were available within this window, the most recently obtained value prior to baseline was used. For follow-up assessments, laboratory values obtained up to one month prior to 90-day follow-up were used. This window was chosen to capture values reflecting the patient's metabolic status at the time of the follow-up clinical assessment. This 90-day interval between OPM study assessments is consistent with the minimum recommended interval (three months) between HbA1c measurements in patients with diabetes [27].

### Definition of control status and clinical inertia

In the OPM study, control status for each condition was defined according to current international guidelines [25–28]:

- Hypertension control: systolic blood pressure (SBP) <130 mmHg and diastolic blood pressure (DBP) <80 mmHg for patients aged <65 years; SBP <140 mmHg and DBP <80 mmHg for patients aged ≥65 years.
- Diabetes control: HbA1c <7.0% for patients aged <80 years; HbA1c <8.0% for patients aged ≥80 years.
- Dyslipidaemia control: LDL-c <70 mg/dL for patients classified as high CVR; LDL-c <55 mg/dL for patients classified as very high CVR.

We defined clinical inertia in accordance with Phillips *et al.* [6] and the OPM study protocol, as the failure of the treating physician to initiate pharmacological treatment, increase dosage, or implement appropriate drug combinations when a cardiovascular risk factor remained uncontrolled, among therapeutically adherent patients who remained in follow-up. Operational criteria were uncontrolled hypertension, diabetes, or dyslipidaemia at baseline per ESC-aligned thresholds above, and no documented pharmacological change over the 90-day follow-up period (code 0) *vs.* modification (code 1) or intensification (code 2). Study procedures record whether change occurred at any point during follow-up, ascertained at study close-out. Clinical inertia was classified according to the study procedures. These variables document whether pharmacological treatment was unchanged, modified, or intensified at any point during the 90-day follow-up period among patients uncontrolled at the index assessment, rather than representing a single-assessment snapshot. In this analysis, clinical inertia was operationalised as:

- Hypertension inertia: uncontrolled blood pressure at baseline with no documented antihypertensive pharmacological change over 90-day follow-up.
- Diabetes inertia: uncontrolled HbA1c at baseline with no documented antidiabetic pharmacological change over 90-day follow-up.
- Dyslipidaemia inertia: uncontrolled LDL-c at baseline with no documented lipid-lowering pharmacological change over 90-day follow-up.
- Any inertia: clinical inertia in at least one of the three conditions.

### Outcomes

Our primary outcomes were prevalence of clinical inertia for each CVR condition, estimated as the proportion of patients with an uncontrolled condition at baseline who did not receive treatment intensification; and factors associated with clinical inertia, including age, sex, CVR category, and excess weight. Our secondary outcome included changes in CVR factor measurements from baseline to 90 days, comparing patients with and without clinical inertia, including SBP and DBP (mmHg), TC, LDL-c, HDL-c, and TG (mg/dL), FPG (mg/dL), HbA1c (%), and BMI (kg/m^2^).

### Statistical analysis

Continuous variables were summarised as medians (Mdn) with interquartile ranges (IQR), given the non-normal distribution of most CVR parameters (confirmed using Shapiro-Wilk testing and visual inspection of histograms). We presented categorical variables as frequencies and percentages. We performed comparisons of baseline characteristics between patients with and without clinical inertia using the Mann-Whitney U test for continuous variables and the χ^2^ test (or Fisher exact test where expected cell counts were <5) for categorical variables.

Furthermore, we calculated the prevalence of clinical inertia for each condition as the proportion of patients with an uncontrolled condition at baseline who did not receive treatment intensification, expressed as a percentage with exact 95% confidence intervals (CIs). We quantified the impact of clinical inertia on persistent uncontrolled status using relative risk (RR), absolute risk difference (ARD), and number needed to harm (NNH). RR represents the ratio of the proportion remaining uncontrolled in the inertia group to that in the no-inertia group. ARD is the arithmetic difference in these proportions (inertia minus no inertia); we calculated NNH as 1/ARD when ARD > 0. We computed 95% CIs using standard methods and assessed statistical significance using the χ^2^ test.

To identify patient-level factors associated with clinical inertia, we fitted multivariable logistic models for each condition and for any inertia. We selected covariates *a priori* based on clinical relevance: age (per 10-year increment), sex (female *vs.* male), CVR category (very high *vs.* high), and excess weight (BMI≥25 kg/m^2^ *vs.* <25 kg/m^2^). Prior to model fitting, we calculated variance inflation factors (VIFs) to assess multicollinearity; all VIFs were <2.0, indicating no problematic collinearity. We assessed calibration using the Hosmer–Lemeshow goodness-of-fit test, and reported model discrimination as Nagelkerke pseudo-R^2^. We expressed results as odds ratios (OR) with 95% CIs.

To evaluate the impact of clinical inertia on longitudinal CVR factor outcomes, we fitted linear mixed-effects models (LMM) for each continuous outcome variable. Models included fixed effects for inertia status (inertia *vs.* no inertia), assessment (baseline *vs.* 90-day follow-up), and their interaction term, with a random intercept per patient to account for within-subject correlation. The interaction term (inertia × assessment) was the primary parameter of interest, representing the differential change from baseline to 90 days between groups. Results were expressed as estimated mean changes (Δ) with 95% CIs within each group, and differences in changes between groups. We assessed model assumptions (normality of residuals, homoscedasticity) by inspecting residual plots.

We conducted baseline comparisons among participants with complete data. We fitted linear mixed-effects models using all available time point observations under a missing-at-random assumption. HbA1c was missing in 7.9% of participants at baseline, and we did not perform imputation. For between-group LMM comparisons, we estimated *post hoc* power from observed interaction effect sizes and pooled standard deviations (SDs).

This study was conducted as a secondary analysis of the OPM study. We determined sample size according to the parent study’s primary objectives. The analytic sample of 711 participants provided adequate coverage for the prevalence estimation aims. For the logistic regression models, the minimum number of outcome events across conditions (dyslipidaemia inertia: n = 217; diabetes inertia: n = 73) satisfied established events-per-variable criteria [29] for the four-covariate models considered. Given the exploratory nature of some secondary analyses, all findings should be interpreted cautiously, with replication in larger, purpose-designed studies encouraged.

We applied a two-sided significance level of α = 0.05 throughout our analysis, but did not adjust *P*-values for multiple comparisons. Sensitivity analyses included dyslipidaemia inertia prevalence using LDL-c <70 mg/dL for all high/very-high-risk patients; and any inertia by age ≥65 years in multivariable logistic models (covariates as in the primary analysis). We performed all analyses using *R*, version 4.4.1 (R Core Team, Vienna, Austria).

## RESULTS

### Participant characteristics

**Figure 1:**
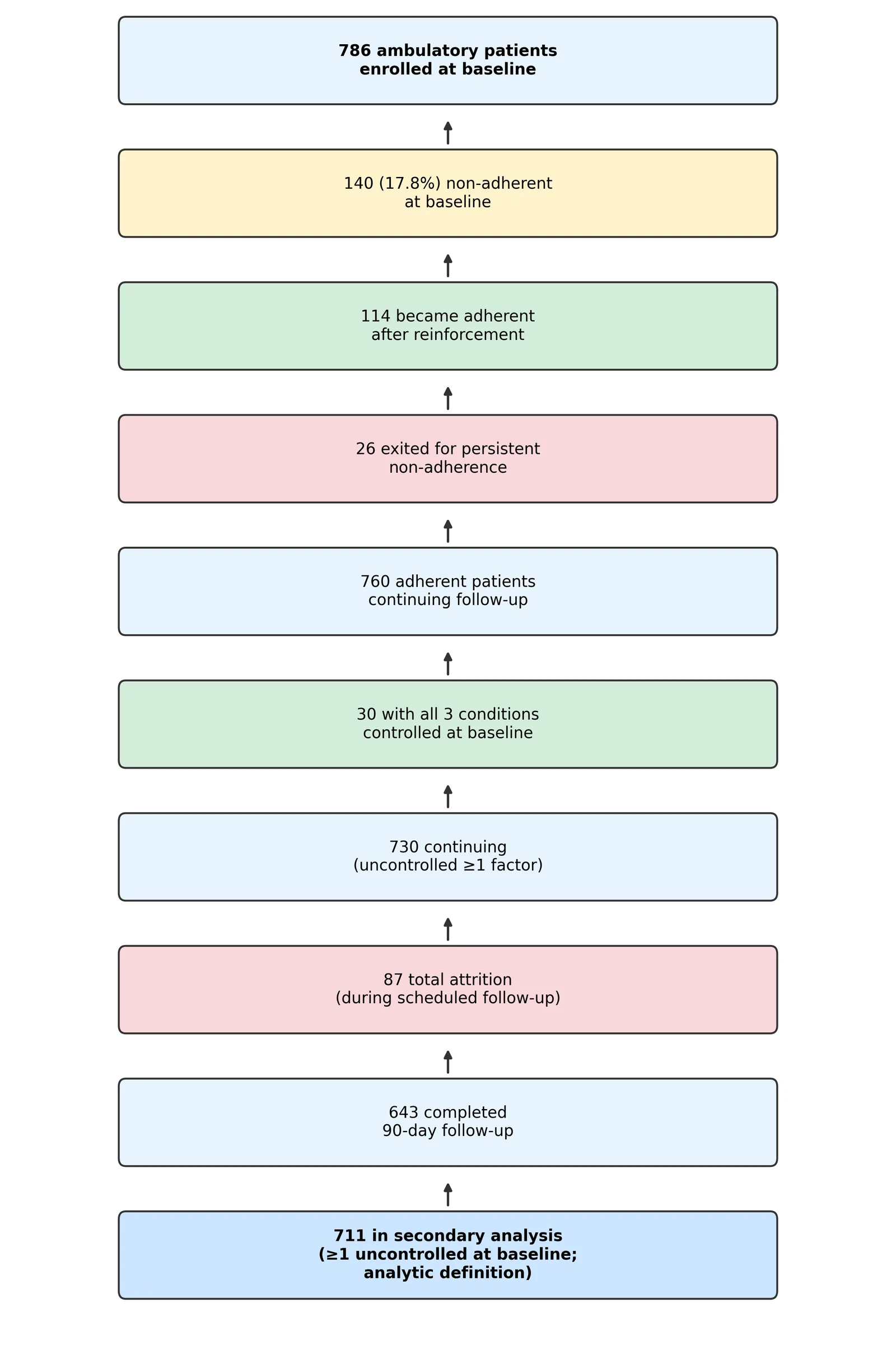
Participant flow for the ambulatory OPM cohort (501 dialysis patients analysed separately and excluded).

A total of 786 ambulatory participants completed the baseline assessment. Of these, 711 (90.5%) had at least one uncontrolled CVR factor at baseline and were included in the present analysis (**Figure 1**).

The Mdn age of the analytic sample was 66.0 years (IQR = 60.0–73.0), and 39.0% (n = 277) were female. Among these 711 participants, 386 (54.3%) exhibited clinical inertia in at least one condition. We observed no statistically significant differences between groups for any baseline variable, including age (*P* = 0.786), sex (*P* = 0.642), BMI (*P* = 0.751), TC (*P* = 0.094), LDL-c (*P* = 0.080), or CVR category (*P* = 0.139), reflecting similar baseline profiles across groups (**Table 1**).

**Table 1:** Baseline characteristics of study participants according to clinical inertia status*

Variable	No inertia (n = 325)	Inertia (n = 386)	*P*-value†
Sex, n (%)			0.642
*Male*	195 (60.0)	239 (61.9)	
*Female*	130 (40.0)	147 (38.1)	
Age in years‡	66.0 (60.0, 73.0)	66.0 (60.0, 74.0)	0.786
SBP (mmHg)	135.0 (125.0, 150.0)	137.0 (129.0, 150.0)	0.213
DBP (mmHg)	79.0 (72.0, 90.0)	80.0 (75.0, 87.0)	0.645
TC (mg/dL)‡	181.0 (151.0, 222.0)	165.5 (139.8, 200.0)	0.094
LDL-c (mg/dL)‡	105.3 (79.8, 144.2)	89.0 (67.0, 115.0)	0.080
HDL-c (mg/dL)‡	49.0 (42.0, 58.0)	47.5 (41.0, 59.0)	0.453
TG (mg/dL)‡	128.5 (96.0, 176.2)	128.5 (92.0, 181.0)	0.719
FPG (mg/dL)	118.0 (95.0, 141.0)	121.0 (98.0, 143.8)	0.327
HbA1c (%)‡	6.6 (6.0, 7.5)	6.7 (5.9, 7.5)	0.881
BMI (kg/m^2^)‡	29.6 (26.7, 32.3)	29.0 (26.6, 32.9)	0.751
Excess weight, n (%)			0.882
*No*	57 (17.5)	66 (17.1)	
*Yes*	268 (82.5)	320 (82.9)	
CVR category, n (%)			0.139
*High*	142 (43.7)	148 (38.3)	
*Very high*	183 (56.3)	238 (61.7)	

BMI – body mass index, CVR – cardiovascular risk, DBP – diastolic blood pressure, FPG – fasting plasma glucose, HbA1c – glycated haemoglobin, HDL-c – high-density lipoprotein cholesterol, IQR – interquartile range, LDL-c – low-density lipoprotein cholesterol, Mdn – median, SBP – systolic blood pressure, TC – total cholesterol, TG – triglycerides

*Presented as Mdn (IQR) unless specified otherwise.

†Mann-Whitney U test for continuous variables; χ^2^ test for categorical variables.

‡Missing data: age, n = 2 (0.3%); TC, LDL-c, HDL-c, TG, n = 4–5 (0.6–0.7%); HbA1c, n = 56 (7.9%); BMI, n = 9 (1.3%).

### Prevalence of clinical inertia and transitions in control status

**Figure 2:**
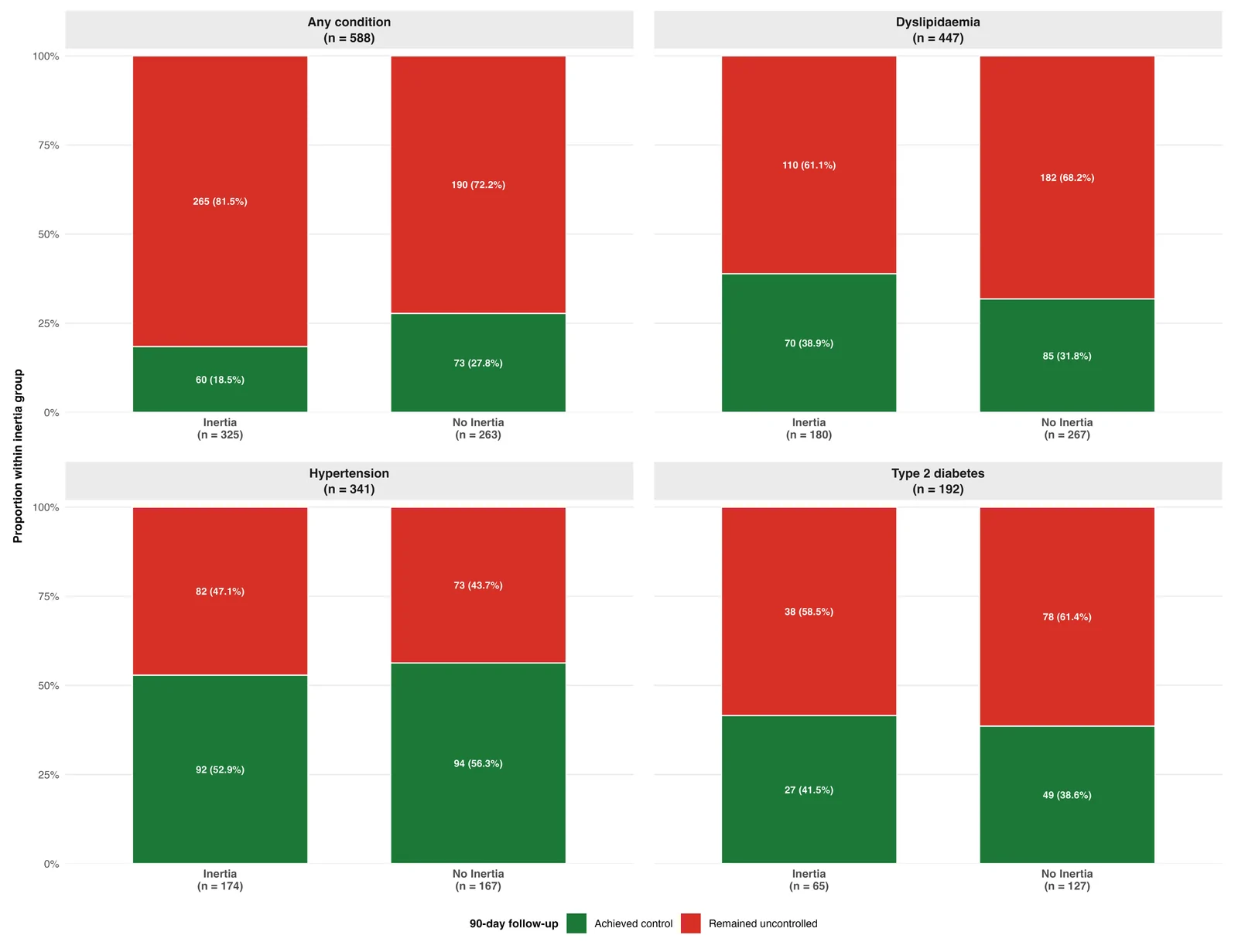
Proportion of patients achieving guideline control at 90-day follow-up among those uncontrolled at baseline, stratified by clinical inertia status (no inertia *vs.* inertia), for hypertension, type 2 diabetes, dyslipidaemia, and any uncontrolled condition. Stacked bars show the composition of 90-day outcomes within each inertia group; segment labels indicate n (%) within group. Green = achieved control, red = remained uncontrolled.

Among patients with an uncontrolled condition at baseline, we observed clinical inertia in 49.3% (n/N = 208/422; 95% CI = 44.7, 53.9) for hypertension, 31.1% (n/N = 73/235; 95% CI = 25.2, 37.5) for diabetes, and 38.2% (n/N = 217/568; 95% CI = 34.3, 42.3) for dyslipidaemia. Among patients with uncontrolled hypertension at baseline, eCRF-documented pharmacological responses were no change in 49.3% (classified as inertia), modification in 30.3%, and intensification in 16.1% (3.8% with missing change codes) (**Figure 2**, **Table 2**).

**Table 2:** Impact of clinical inertia on persistent uncontrolled status: risk analysis

Condition*	No inertia: remained uncontrolled, n/N (%)	Inertia: remained uncontrolled, n/N (%)	RR (95% CI)†	ARD (95% CI)‡
Hypertension	73/167 (43.7)	82/174 (47.1)	1.08 (0.85, 1.36)	3.4% (−7.2, 14.0)
Type 2 diabetes	78/127 (61.4)	38/65 (58.5)	0.95 (0.74, 1.22)	−3.0% (−17.6, 11.7)
Dyslipidaemia	182/267 (68.2)	110/180 (61.1)	0.90 (0.78, 1.03)	−7.1% (−16.1, 2.0)
Any condition	190/263 (72.2)	265/325 (81.5)	1.13 (1.03, 1.24)§	9.3% (2.4, 16.2)

ARD – absolute risk difference (inertia minus no inertia), CI – confidence interval, NNH – number needed to harm, RR – relative risk

*Analysis restricted to patients with at least one uncontrolled CVR factor at baseline. Denominators reflect patients with the specific uncontrolled condition at baseline and available 90-day control assessment data.

†RR>1 indicates that clinical inertia increases the risk of remaining uncontrolled.

‡NNH is calculated only when ARD > 0 (any condition: NNH ≈ 11).

§*P* < 0.05.

We compared rates of remaining uncontrolled at 90-day follow-up between patients with and without inertia at baseline to quantify the impact of clinical inertia on persistent uncontrolled status. For hypertension, 73/167 (43.7%) without inertia remained uncontrolled, compared with 82/174 (47.1%) with inertia (RR = 1.08; 95% CI = 0.85, 1.36; ARD = 3.4%; 95% CI = −7.2, 14.0, *P* = 0.527). For diabetes, 78/127 (61.4%) without inertia remained uncontrolled *vs.* 38/65 (58.5%) with inertia (RR = 0.95; 95% CI = 0.74–1.22; ARD = −3.0%; 95% CI = −17.6, 11.7, *P* = 0.692). For dyslipidaemia, 182/267 (68.2%) without inertia remained uncontrolled *vs.* 110/180 (61.1%) with inertia (RR = 0.90; 95% CI = 0.78, 1.03; ARD = −7.1%; 95% CI = −16.1, 2.0, *P* = 0.124). None of these condition-specific comparisons reached statistical significance.

We observed a statistically significant difference when we performed a pooled analysis across all conditions (patients with at least one uncontrolled CVR factor at baseline): 190/263 (72.2%) without clinical inertia remained uncontrolled in at least one condition at follow-up, compared with 265/325 (81.5%) with clinical inertia (RR = 1.13; 95% CI = 1.03, 1.24; ARD = 9.3%; 95% CI = 2.4, 16.2, *P* = 0.007; NNH ≈ 11).

### Factors associated with clinical inertia

For any inertia (at least one condition), we found no statistically significant associations with age (OR per 10 years = 1.02; 95% CI = 0.88, 1.18, *P* = 0.781), sex (OR for female *vs.* male = 0.96; 95% CI = 0.70, 1.30, *P* = 0.773), CVR category (OR for very high *vs.* high = 1.25; 95% CI = 0.91, 1.70, *P* = 0.162), or excess weight (OR = 0.99; 95% CI = 0.67, 1.47, *P* = 0.966). Similarly, we observed non-significant patterns in condition-specific models. The Hosmer–Lemeshow test indicated adequate model calibration for all fitted models (all *P* > 0.05). Nagelkerke pseudo-R^2^ values ranged from 0.001 to 0.014, reflecting the negligible explanatory capacity of the included patient-level factors. VIFs were below 2.0 for all predictors, confirming absence of multicollinearity (**Table 3**).

**Table 3:** Factors associated with clinical inertia: multivariable logistic regression

	OR (95% CI)*	*P*-value
**Any condition**		
Age (per 10 years)	1.02 (0.88, 1.18)	0.781
CVR category (very high *vs.* high)	1.25 (0.91, 1.70)	0.162
Excess weight (yes *vs.* no)	0.99 (0.67, 1.47)	0.966
Sex (female *vs.* male)	0.96 (0.70, 1.30)	0.773
Hosmer–Lemeshow *P*-value	0.713	
Nagelkerke R^2^	0.005	
**Hypertension**		
Age (per 10 years)	0.96 (0.79, 1.17)	0.707
CVR category (very high *vs.* high)	1.37 (0.90, 2.08)	0.141
Excess weight (yes *vs.* no)	0.75 (0.42, 1.33)	0.329
Sex (female *vs.* male)	1.11 (0.73, 1.69)	0.639
Hosmer–Lemeshow *P*-value	0.586	
Nagelkerke R^2^	0.010	
**Type 2 diabetes**		
Age (per 10 years)	0.90 (0.66, 1.23)	0.504
CVR category (very high *vs.* high)	1.01 (0.56, 1.84)	0.982
Excess weight (yes *vs.* no)	0.96 (0.46, 2.05)	0.907
Sex (female *vs.* male)	0.82 (0.45, 1.47)	0.503
Hosmer–Lemeshow *P*-value	0.812	
Nagelkerke R^2^	0.006	
**Dyslipidaemia**		
Age (per 10 years)	1.15 (0.97, 1.35)	0.107
CVR category (very high *vs.* high)	1.06 (0.74, 1.52)	0.767
Excess weight (yes *vs.* no)	0.88 (0.55, 1.41)	0.592
Sex (female *vs.* male)	0.79 (0.55, 1.14)	0.207
Hosmer–Lemeshow *P*-value	0.634	
Nagelkerke R^2^	0.014	

CI – confidence interval, CVR – cardiovascular risk, OR – odds ratio

*Reference categories: CVR category (high), excess weight (no), sex (male). All models adjusted for age, sex, CVR category, and excess weight

### Effect of clinical inertia on cardiovascular risk factor outcomes

Both groups demonstrated statistically significant reductions in blood pressure, lipid parameters, FPG, and BMI over the 90-day follow-up. However, for lipid outcomes, the magnitude of improvement differed substantially between groups. Patients without clinical inertia showed markedly larger reductions in TC (−33.9 mg/dL; 95% CI = −40.0, −27.7, *P* < 0.001) compared with patients with inertia (−15.0 mg/dL; 95% CI = −18.8, −11.2, *P* < 0.001), yielding a statistically significant and clinically meaningful between-group difference of 19.3 mg/dL (model-based) (95% CI = 12.4, 26.3, *P* < 0.001) in favour of the no-inertia group. We observed an analogous pattern for LDL-c (−30.4 mg/dL without inertia *vs.* −15.1 mg/dL with inertia), with the between-group difference reaching 15.5 mg/dL (95% CI = 8.9, 22.0, *P* < 0.001), reflecting substantially greater LDL-c reduction among patients who received treatment intensification. For blood pressure, both groups showed significant within-group reductions; the between-group difference in magnitude of change was not statistically significant (Table S3 in the **Online Supplementary Document**).

We also found a small but statistically significant between-group difference in BMI reduction (0.25 kg/m^2^; 95% CI = 0.02, 0.49, *P* = 0.036), with patients without inertia achieving modestly greater weight loss. We observed no statistically significant between-group differences for SBP, DBP, HDL-c, TG, FPG, or HbA1c (Figure S1 in the **Online Supplementary Document**).

### *Post hoc* power

For between-group LMM comparisons, *post hoc* power estimated from observed interaction effect sizes and pooled SDs was approximately 74% for LDL-c (observed between-group difference ≈ 15.5 mg/dL) and less than 20% for SBP (Δ ≈ 0.3 mmHg) and HbA1c (Δ ≈ 0.13 percentage points). Condition-specific analyses of persistent uncontrolled status were underpowered to detect small relative risk differences (*e.g.* hypertension RR = 1.08).

### Sensitivity analyses

In a sensitivity analysis applying LDL-c <70 mg/dL for all high- and very-high-risk patients, dyslipidaemia inertia prevalence was 37.5% (n/N = 191/509) compared with 38.2% (n/N = 217/568) using ESC-stratified thresholds in the primary analysis; estimates were stable in direction. When age was modelled categorically (≥65 *vs.* <65 years) in the multivariable logistic model for any inertia, age was not associated with clinical inertia (OR = 0.85; 95% CI = 0.58, 1.24, *P* = 0.40), consistent with null findings for age per 10-year increment (**Table 3**).

## DISCUSSION

This multicentre, real-world study provides comprehensive evidence on the prevalence, determinants, and consequences of clinical inertia in high-risk primary care patients with uncontrolled CVR factors. Three overarching findings emerged. First, clinical inertia is highly prevalent, affecting more than half of the study population across the three major modifiable CVR factors. Second, patient-level characteristics, including age, sex, CVR category, and excess weight, were not meaningfully associated with inertia in this sample. This pattern is consistent with, but does not prove, provider- and system-level influences described in recent real-world primary care studies [30–37]; physician- and centre-level variables were not available for direct testing. Third, clinical inertia has quantifiable consequences: patients who did not receive treatment intensification showed substantially smaller reductions in total and LDL cholesterol over 90 days, with implications for long-term cardiovascular risk.

The proportions observed in this study (49% for hypertension, 31% for diabetes, and 38% for dyslipidaemia) are broadly consistent with published literature. Our estimate falls at the upper end of the 30–50% range typically reported [11,12,34,35,38] for hypertension, which may reflect the pragmatic, routine-care context of the OPM study, where time constraints and competing clinical demands are greater than in trial settings. Inertia rates of 31% for diabetes and 38% for dyslipidaemia align well with ranges reported in prior observational and recent systematic reviews [13–16,36,39,40].

The absence of any statistically significant association between clinical inertia and patient-level factors has substantial conceptual and practical importance. The tested variables (*i.e.* age, sex, CVR category, and excess weight) represent commonly cited patient characteristics that might be expected to influence physicians’ treatment decisions. Their uniformly negligible ORs, combined with near-zero Nagelkerke pseudo-R2 values (0.001–0.014), indicate that inertia is not selectively concentrated in any identifiable patient subgroup. This is consistent with results from some, though not all, prior studies [41,42], and suggests that interventions targeting specific patient profiles are unlikely to be effective.

Importantly, the patient characteristics examined in this study reflect only one dimension of the complex clinical encounter. The patient-level perspective on therapeutic inertia is frequently underappreciated. In real-world practice, many instances of apparent clinical inertia may reflect not physician passivity but active patient resistance or preference. Patients may decline treatment intensification because they believe additional medications are unnecessary or harmful, because they hold strong preferences for lifestyle modification before pharmacotherapy, because they have experienced or fear side effects from prior treatments, or because psychosocial factors, including health anxiety, low trust in healthcare providers, or limited health literacy, create barriers to acceptance [5,43–45]. Treatment non-adherence further complicates the picture; for example, a physician who recognises that a patient has consistently not taken prescribed medications may reasonably defer intensification pending improved adherence, rendering an apparent inertia decision clinically appropriate [46]. Our study design did not capture these patient-reported reasons for non-intensification, and the variables examined (age, sex, BMI, CVR category) cannot serve as proxies for these attitudes and beliefs. Future studies should incorporate patient-reported outcome measures, health beliefs questionnaires, and structured adherence assessments to disentangle true physician-driven inertia from patient-mediated barriers to intensification.

The impact of clinical inertia on lipid outcomes over 90 days deserves careful interpretation. Patients without inertia achieved substantially greater reductions in TC (−33.9 mg/dL) and LDL-c (−30.4 mg/dL), compared with −15.0 mg/dL and −15.1 mg/dL in the inertia group, yielding between-group differences of 19.3 mg/dL for TC and 15.5 mg/dL for LDL-c. These differences are clinically meaningful. Based on Cholesterol Treatment Trialists’ meta-analyses, each 1 mmol/L (≈ 39 mg/dL) reduction in LDL-c is associated with an approximately 22% reduction in major vascular events [47]. While the 15.5 mg/dL LDL-c difference observed here corresponds to approximately 0.4 mmol/L, it occurs against a backdrop of already high CVR in this population, and the cumulative effect of sustained failure to intensify lipid-lowering therapy over years (as opposed to 90 days) could translate into substantially higher cardiovascular event rates.

The absence of significant between-group differences for blood pressure and glycaemic parameters at 90 days warrants careful consideration rather than dismissal. Several non-mutually-exclusive explanations are plausible. Regression to the mean may have attenuated detectable differences, since both groups started with uncontrolled values and experienced some natural return toward target. The relatively short 90-day follow-up may be insufficient to capture the full pharmacological impact of antihypertensive or antidiabetic treatment intensification, particularly for medications with delayed onset of action. Additionally, lifestyle changes, improved medication adherence, and carryover effects of ongoing therapies may have produced similar effects in both groups for these parameters, masking the incremental benefit of formal treatment intensification. The 90-day window may be better powered to detect lipid changes, which are more immediate consequences of statin dose adjustment, than blood pressure or glycaemic changes.

Clinical inertia also significantly increased the risk of remaining uncontrolled in at least one CVR factor at 90 days (RR = 1.13; NNH ≈ 11). The condition-specific analyses did not reach statistical significance, likely due to insufficient power within each subgroup. Therefore, the overall finding should be interpreted as a broad signal of cumulative risk persistence rather than condition-specific failure.

### Limitations

Several limitations should be considered when interpreting these findings. Patient-level centre and physician identifiers were unavailable. We cannot exclude unmeasured centre-level heterogeneity despite restriction to ambulatory family medicine and internal medicine settings. The parent interventional protocol with structured follow-up may have underestimated inertia prevalence (Hawthorne effect). Pharmacological change codes (0/1/2) do not verify guideline-concordance of intensification.

As a secondary analysis of the OPM interventional study, the original design was optimised for the parent study’s primary objectives rather than specifically for the assessment of clinical inertia. In the OPM study, participating physicians were enrolled on a consecutive basis to minimise selection bias; however, the quasi-experimental nature of the parent study may have introduced selection effects that are difficult to fully characterise. In particular, the study population comprised patients already known to have uncontrolled CVR factors, and findings may not generalise to all primary care patients.

The 90-day follow-up period limits our ability to assess the sustained or cumulative impact of clinical inertia. While we observed meaningful short-term differences in lipid outcomes, the longer-term trajectory of patients who experienced inertia, and whether delayed intensification eventually closes the gap, remains unknown. The absence of significant differences in blood pressure and glycaemic outcomes at 90 days should not be interpreted as absence of harm. These effects may require a longer observation window to become apparent, and this interpretation is itself a hypothesis requiring prospective testing.

We did not capture physicians’ rationale for not intensifying treatment, which is a fundamental limitation when interpreting any operationalised inertia measure. What is classified as clinical inertia in our analysis may include clinically justified decisions. For example, deferring intensification because a patient had recently started a new medication, because adherence to existing therapy was poor, because a shared decision-making process led to a lifestyle-first approach, or because the physician judged the absolute CVR benefit to be marginal relative to the burden of polypharmacy. Without access to this clinical reasoning, the estimated inertia prevalence may overstate the true rate of unjustified therapeutic passivity. Future studies should use structured physician questionnaires or electronic health record-derived decision flags to distinguish justifiable from avoidable inertia.

Furthermore, we based our definition of clinical inertia exclusively on documented pharmacological treatment changes. Non-pharmacological interventions (*e.g.* dietary modification, structured exercise programmes, referral to nurse-led management) that may have been implemented but not captured in the EMR could confound the association between apparent inertia and outcomes. If patients in the inertia group received intensive lifestyle counselling that was not recorded, the observed between-group differences in lipid outcomes might underestimate the true effect of pharmacological inertia.

The use of diabetes duration >10 years as a proxy criterion for target organ damage, in accordance with 2021 ESC guideline criteria [25], deserves specific acknowledgement. Long-standing diabetes does not, by itself, confirm the presence of microvascular or macrovascular complications: a substantial proportion of patients with >10 years of diabetes remain complication-free, and conversely, some patients with shorter duration may already have subclinical nephropathy or retinopathy. Duration of diabetes was ascertained from the EMR diagnosis date, which may not perfectly reflect the actual onset of metabolic dysregulation. This misclassification likely introduces non-differential bias in the ascertainment of CVR category, the magnitude of which cannot be determined from available data. Future studies should incorporate systematic assessment of target organ damage (albuminuria, eGFR, retinal imaging) rather than relying on disease duration.

The study was conducted exclusively in nine Spanish regions, within a public primary care system. It is uncertain to which degree these findings apply to other healthcare systems, with different financing models, prescribing constraints, patient expectations, or physician training. Spain’s primary care context, which is characterised by relatively long-established primary care infrastructure and universal health coverage, may not reflect conditions in other high-income or low-to-middle-income countries.

Finally, genuinely treatment-resistant conditions (*e.g.* refractory hypertension, statin-refractory hypercholesterolaemia) cannot be distinguished in the eCRF; such patients may have been classified as clinical inertia when non-intensification reflected appropriate clinical judgment, plausibly inflating prevalence estimates in an unquantifiable direction.

### Future research directions

Building on these findings, several research priorities emerge. Studies with longer follow-up periods (at least 12–24 months) are needed to assess whether clinical inertia produces sustained differences in CVR factor control and, ultimately, in hard cardiovascular endpoints. Qualitative and mixed-methods studies that elicit physicians’ and patients’ perspectives on treatment intensification decisions would provide crucial mechanistic insight, enabling more targeted interventions. Future studies should expand the patient-level variable set to include health beliefs, medication adherence measures, patient–physician communication quality, and psychosocial determinants. Multi-country studies are needed to evaluate whether the determinants and consequences of clinical inertia are generalisable across different healthcare systems and cultural contexts. Finally, interventional research testing specific de-implementation strategies (*e.g.* clinical decision support systems, performance feedback dashboards, structured quality improvement cycles) [48,49] should prioritise outcomes that capture both process (inertia rates) and patient-level endpoints (CVR factor control, cardiovascular events).

## CONCLUSIONS

Clinical inertia is pervasive among high-risk primary care patients with uncontrolled CVR factors, affecting more than half of this population simultaneously across hypertension, diabetes, and dyslipidaemia. The uniformly negligible associations between inertia and patient-level characteristics point to systemic and provider-level drivers, arguing for interventions that target the clinical encounter, decision-support infrastructure, and quality improvement systems rather than specific patient subgroups.

The demonstrably smaller improvements in total and LDL cholesterol observed over 90 days among patients who did not receive treatment intensification are clinically consequential: even short-term lipid differences of this magnitude, in patients already at high cardiovascular risk, could translate into meaningful increases in long-term event rates. Addressing clinical inertia is therefore not merely a process quality metric; it is a patient safety issue with direct implications for cardiovascular prevention.

## Additional material


Online Supplementary Document

## Data Availability

**Data availability:** The generated and analysed data sets are available from the corresponding author upon reasonable request.
