# Supplementary material for: Clinical inertia in cardiovascular risk management: prevalence, associated factors, and impact on outcomes of the OPM study
Source: J Glob Health. 2026 Sep 11;16:04256. doi: 10.7189/jogh.16.04256 (PMC13564932; doi:10.7189/jogh.16.04256)
Supplement: Supplementary file 1 [file jogh-16-04256-s001.pdf]

Supplement to: Abellán Alemán J, Sánchez-Rubio Lezcano P, Escribano Pardo D, Castilla Guerra L, Crespo Sabaris R, Jiménez Portillo G, Rincón Ruiz B, Antón Pérez G, Iturralde Iriso J, Segura P, Tárraga López P, Nieto Iglesias J, López-Gil J; on behalf of the researchers of the OPM study. Clinical inertia in cardiovascular risk management: prevalence, associated factors, and impact on outcomes of the OPM study. J Glob Health. 2026;16:04256.

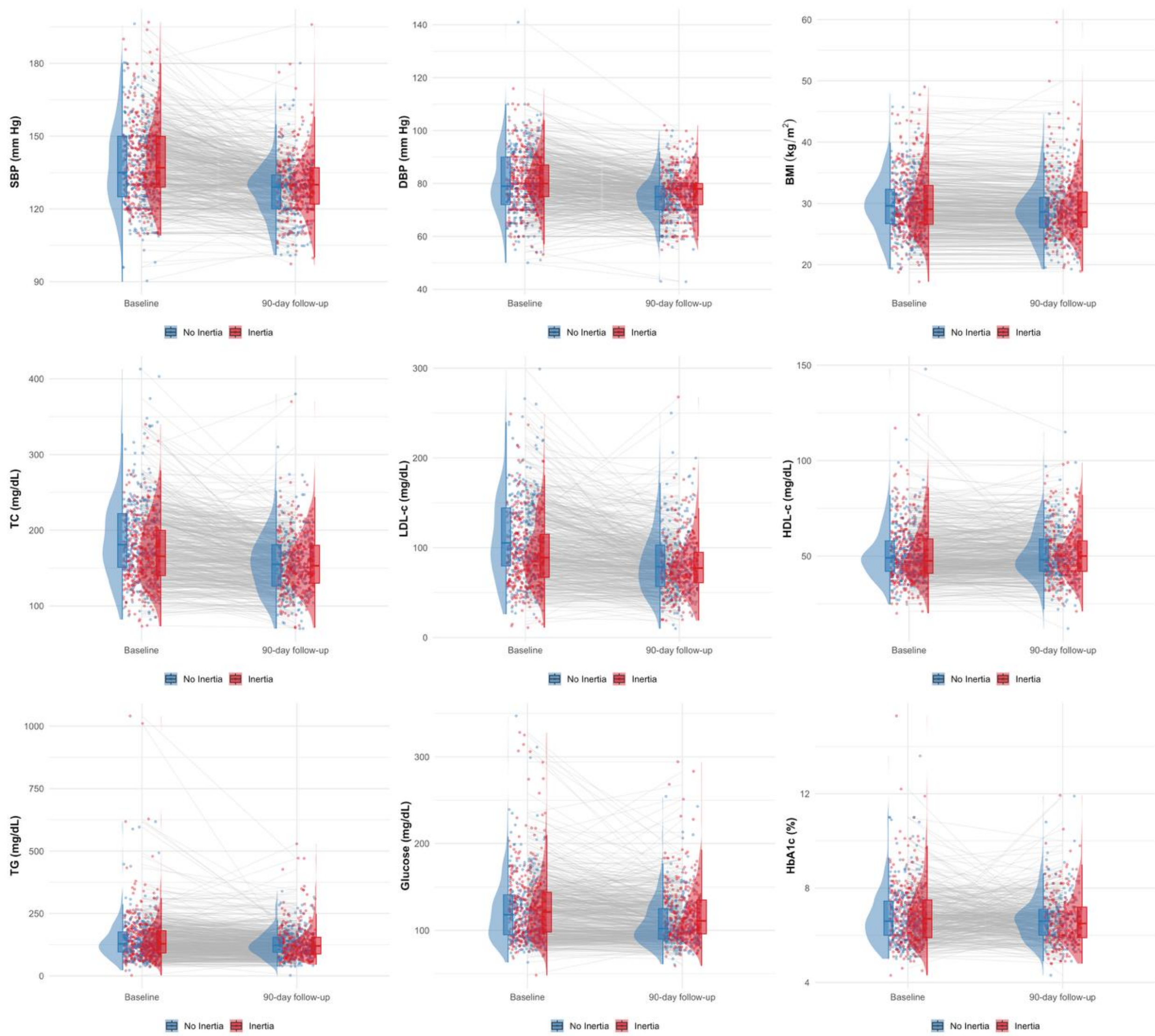

Figure S1.
Distribution of changes in cardiovascular risk factors from baseline to 90 days according to clinical inertia status. Box plots show median and interquartile range; whiskers extend to 1.5×IQR. Positive values indicate increases; negative values indicate reductions from baseline.

Table S1.
Completed STROBE checklist.

**Title of manuscript**	Clinical inertia in cardiovascular risk management: prevalence, associated factors, and impact on outcomes: The OPM study
**Authors**	Abellán Alemán J, et al.
**Journal**	Journal of Global Health (Article 13333)
**Study design (for checklist)**	Secondary analysis of a prospective cohort (ambulatory OPM study); quasi-experimental parent trial

**Section**	**Item**	**Recommendation**	**Reported on page / location**
Title and abstract	1a	Indicate the study's design with a commonly used term in the title or the abstract	Abstract, p.1; Methods (secondary analysis; prospective cohort)
Title and abstract	1b	Provide in the abstract an informative and balanced summary of what was done and what was found	Abstract (Background, Methods, Results, Conclusions)
Introduction	2	Explain the scientific background and rationale for the investigation being reported	Introduction, pp.2–3
Introduction	3	State specific objectives, including any prespecified hypotheses	Introduction (final paragraph); Methods (Primary/secondary outcomes)
Methods	4	Present key elements of study design early in the paper	Methods — Study design and setting; Parent OPM study intervention
Methods	5	Describe the setting, locations, and relevant dates, including periods of recruitment, exposure, follow-up, and data collection	Methods — Setting (9 Spanish regions, 2024–2025); Study visits (V1–V4, 90-day follow-up)
Methods	6a	Cohort study — eligibility criteria, sources and methods	Methods — Patient recruitment and eligibility; Parent

		of selection, methods of follow-up	OPM intervention; Figure S2
Methods	6b	Cohort study — matching criteria (if matched study)	Not applicable (unmatched cohort)
Methods	7	Clearly define all outcomes, exposures, predictors, potential confounders, and effect modifiers	Methods — Definition of control status and clinical inertia; Outcomes; Covariates (age, sex, CVR category, excess weight)
Methods	8	For each variable of interest, give sources of data and details of methods of assessment	Methods — Study visits and data collection; Clinical measurements (BP, labs, eCRF vs EMR)
Methods	9	Describe any efforts to address potential sources of bias	Methods — Adherence ascertainment/exclusion; Parent intervention/Hawthorne discussion; Limitations
Methods	10	Explain how the study size was arrived at	Methods — Statistical analysis (711 analytic sample; EPV for logistic regression; parent trial size)
Methods	11	Explain how quantitative variables were handled in the analyses	Methods — Statistical analysis (medians/IQR; age per 10 years; BMI ≥25)
Methods	12a	Describe all statistical methods, including those used to control for confounding	Methods — Statistical analysis (logistic regression, LMM, RR/ARD/NNH)
Methods	12b	Describe any methods used to examine subgroups and interactions	Methods — LMM Visit × Inertia interaction; condition-specific models
Methods	12c	Explain how missing data were addressed	Methods — Statistical analysis (Missing data paragraph); Table 1 footnote

Methods	12d	Cohort study — explain how loss to follow-up was addressed	Methods — Figure S2; Limitations; complete-case/LMM under MAR
Methods	12e	Describe any sensitivity analyses	Methods/Supplement — LDL <70 mg/dL threshold; age ≥65 years (Supplementary Tables S4–S5)
Results	13a	Report numbers of individuals at each stage of study	Results — Participant characteristics; Figure S2; Supplementary Material
Results	13b	Give reasons for non-participation at each stage	Figure S2 legend (non-adherence exit n=26; attrition n=87 with reasons)
Results	13c	Consider use of a flow diagram	Figure S2 (ambulatory participant flow)
Results	14a	Give characteristics of study participants and information on exposures and confounders	Results — Table 1
Results	14b	Indicate number of participants with missing data for each variable of interest	Table 1 footnote (age, lipids, HbA1c, BMI)
Results	14c	Cohort study — summarise follow-up time	Methods/Results — 90-day follow-up (Visit 1 to Visit 4)
Results	15	Cohort study — report numbers of outcome events or summary measures over time	Results — Table 2 (persistent uncontrolled status); Table 4 (LMM changes V1–V4)
Results	16a	Give unadjusted and confounder-adjusted estimates and their precision	Results — Table 2 (RR, ARD); Table 3 (OR, 95% CI); Table 4 (LMM estimates)
Results	16b	Report category boundaries when continuous variables were categorized	Methods — Control thresholds (BP by age, LDL by CVR, HbA1c by age); age per 10 years
Results	16c	If relevant, translate relative risk into absolute risk for a	Results — Table 2 (ARD, NNH ≈ 11 for any condition)

		meaningful time period	
Results	17	Report other analyses — subgroups, interactions, sensitivity analyses	Supplementary Tables S3–S5; post-hoc power; LDL/age sensitivity
Discussion	18	Summarise key results with reference to study objectives	Discussion (opening paragraphs); Conclusions
Discussion	19	Discuss limitations, sources of potential bias, direction and magnitude	Limitations (entire section)
Discussion	20	Give a cautious overall interpretation considering objectives, limitations, and prior evidence	Discussion; Conclusions (revised cautious wording)
Discussion	21	Discuss the generalisability (external validity) of the study results	Limitations/Discussion (Spain, ambulatory MF/MI, high-risk selection)
Other information	22	Give the source of funding and the role of the funders	Funding statement: Not applicable

Note: This checklist was completed for the secondary analysis of clinical inertia in the OPM study. Cohort-specific items apply where relevant; cross-sectional and case-control–specific sub-items are marked 'Not applicable' when appropriate. An Explanation and Elaboration document is available at www.strobe-statement.org.

Table S2. Research ethics committees approving the OPM study.

Region	Committee	Approval date
Alicante	Research Ethics Committee, Hospital Universitario San Juan de Alicante	28 Sep 2023
Valencia	Ethics Committee for Drug Research, Hospital Clínico Universitario de Valencia	17 Jan 2024
Navarra	Clinical Research Ethics Committee of Navarra	23 Oct 2023
Euskadi	Ethics Committee for Drug Research of Euskadi, Vitoria-Gasteiz	7 Jun 2023
Ciudad Real	Research Ethics Committee for Drug Research, SESCAM, Ciudad Real	18 Jul 2023
Murcia	Medical Research Ethics Committee, Hospital Clínico Universitario Virgen de la Arrixaca	30 May 2023
La Rioja	Research Ethics Committee for Drug Research of La Rioja	10 Oct 2023
Salamanca	Research Ethics Committee for Drug Research, CAU Salamanca	22 May 2023
Balearic Islands	Research Commission, Technical Office of Primary Care	Mar 2023

Table S3. Impact of clinical inertia on cardiovascular risk factor outcomes: linear mixed-effects models

**Outcome**	**No inertia (n = 325)**		**Inertia (n = 386)**			
	**Within-group difference**		**Within-group difference**		**Between-group difference**	
	**Δ (95% CI)***	***P*-value**	**Δ (95% CI)***	***P*-value**	**Δ (95% CI)***	***P*-value**
SBP (mmHg)	−9.98 (−11.70, −8.26)	<0.001	−9.63 (−11.27, −7.99)	<0.001	0.33 (−2.05, 2.72)	0.783
DBP (mmHg)	−5.47 (−6.63, −4.30)	<0.001	−4.58 (−5.56, −3.59)	<0.001	0.90 (−0.62, 2.41)	0.245

TC (mg/dL)	−33.86 (−40.04, −27.68)	<0.001	−15.00 (−18.80, −11.21)	<0.001	19.34 (12.37, 26.30)	<0.001
LDL-c (mg/dL)	−30.36 (−35.91, −24.82)	<0.001	−15.10 (−18.96, −11.24)	<0.001	15.45 (8.87, 22.04)	<0.001
HDL-c (mg/dL)	−0.83 (−2.11, 0.45)	0.201	0.08 (−1.16, 1.32)	0.901	0.90 (−0.89, 2.69)	0.325
TG (mg/dL)	−19.08 (−26.99, −11.17)	<0.001	−16.32 (−25.97, −6.66)	<0.001	2.58 (−10.28, 15.44)	0.694
FPG (mg/dL)	−12.20 (−15.71, −8.68)	<0.001	−9.24 (−13.14, −5.35)	<0.001	3.03 (−2.33, 8.40)	0.268
HbA1c (%)	−0.10 (−0.29, 0.10)	0.327	−0.23 (−0.42, −0.03)	0.021	−0.13 (−0.41, 0.15)	0.355
BMI (kg/m^2)	−0.64 (−0.80, −0.49)	<0.001	−0.39 (−0.57, −0.22)	<0.001	0.25 (0.02, 0.49)	0.036

BMI – body mass index, DBP – diastolic blood pressure, FPG – fasting plasma glucose, HbA1c – glycated haemoglobin, HDL-c – high-density lipoprotein cholesterol, LDL-c – low-density lipoprotein cholesterol, LMM – linear mixed-effects model, SBP – systolic blood pressure, TC – total cholesterol, TG – triglycerides

*Δ = estimated mean change from baseline to 90 days based on LMM. Between-group difference = interaction term (inertia × assessment) from LMM; a positive value indicates that the no-inertia group achieved a greater reduction than the inertia group.
